# Vowel Length Expands Perceptual and Emotional Evaluations in Written Japanese Sound-Symbolic Words

**DOI:** 10.3390/bs11060090

**Published:** 2021-06-21

**Authors:** Zihan Lin, Nan Wang, Yan Yan, Toshimune Kambara

**Affiliations:** Department of Psychology, Graduate School of Humanities and Social Sciences, Hiroshima 7398524, Japan; zihanlin51@gmail.com (Z.L.); 1151608137wn@gmail.com (N.W.); yanyan201904@gmail.com (Y.Y.)

**Keywords:** Japanese, sound symbolism, vowel length, semantic differential scale, written sound-symbolic words, perceptual and emotional evaluations

## Abstract

In this study, we examined whether vowel length affected the perceptual and emotional evaluations of Japanese sound-symbolic words. The perceptual and emotional features of Japanese sound-symbolic words, which included short and long vowels, were evaluated by 209 native Japanese speakers. The results showed that subjective evaluations of familiarity, visual imageability, auditory imageability, tactile imageability, emotional valence, arousal, and length were significantly higher for sound-symbolic words with long vowels compared to those with short vowels. Additionally, a subjective evaluation of speed was significantly higher for written Japanese sound-symbolic words with short vowels than for those with long vowels. The current findings suggest that vowel length in written Japanese sound-symbolic words increases the perceptually and emotionally subjective evaluations of Japanese sound-symbolic words.

## 1. Introduction

In sound symbolism, linguistic features non-arbitrarily associate with perceptual or emotional features [1,2,3,4,5]. For example, a pseudoword including a vowel “*a*” is associated with a bigger figure than a pseudoword including a vowel “*i*” [5]. Previous studies of the *bouba-kiki* effect showed that some pseudowords (e.g., *bouba*) were linked with round figures, whereas other pseudowords (e.g., *kiki*) were linked with spiky figures [6,7]. One study reported that auditory features of spoken pseudowords cross-modal associated with visual features of figures [8]. Linguistic features (specific consonants and vowels) can increase the expected gustatory features [9]. Emotional features are also connected to linguistic information [10]. In addition to sound-symbolic associations between pseudowords (new words) and referents, real words can be sound symbolically associated with perceptual or abstract features [11,12]. Previous findings suggested that the oral shapes to pronounce linguistic features were associated with perceptual and emotional features [5,13], whereas written linguistic features (alphabetic letters and other characters) were also associated with them [14,15,16]. Namba and Kambara (2020) reported that the oral shapes to produce Japanese vowels were connected to specific perceptual and emotional evaluations (e.g., some oral shapes were perceived as bigger, wider, and higher than oral shapes used to produce other vowels) [13]. In addition, Cuskley et al. (2017) showed that pseudowords with round letters (e.g., *gege*) were associated with round figures, while those with spiky letters (e.g., *zeze*) were associated with spiky figures [15]. Based on these previous findings, combinations of spoken and written linguistic features could affect perceptual and emotional sensitivity. Participants learned the sound-symbolic relationships between linguistic features and perceptual features in a first or second language ([17,18,19,20]; see for review [21]), and also learned non-sound-symbolic (arbitrary) relationships between linguistic features and perceptual features in a first or second language [22,23,24,25,26]. For example, event-related brain negativity occurred around 400 ms when infants detected mismatched pairs of linguistic features and figures, compared to matched pairs of linguistic features and figures [17]. Sound-symbolic relationships could occur from already acquired associations between linguistic and referential features in native languages [27]. Other studies also reported that linguistic information was specifically associated with perceptually and emotionally subjective evaluations by using semantic differential scales [14,28,29,30]. For example, participants differentially evaluated familiarity, visual imageability, auditory imageability, tactile imageability, emotional valence (preference), and arousal (excitement) of sound-symbolic words including voiced consonants and sound-symbolic words including semi-voiced or unvoiced consonants [28]. These findings of sound symbolism suggested that linguistic information (written and spoken information) was non-arbitrarily associated with specific perceptual and emotional features.

Previous studies have examined specific associations between perceptual and emotional features of words or pseudowords that included a specific vowel [14,31,32,33]. The vowels of the low second formant (F2) are *a*, *u*, and *o*, whereas vowels of the high F2 are *i* and *e* [31,33,34]. Vowels with low F2 are back vowels, whereas vowels with high F2 are front vowels [31]. The front vowels are associated with smaller features than the back vowels [3,5,35,36]. Such associations between acoustic frequency and referential size are called frequency codes [33]. Klink (2000) reported that products associated with names which included front vowels were evaluated as lighter (relative to both darker and heavier), smaller, more feminine, colder, faster, softer, thinner, friendlier, weaker, prettier, more bitter, and milder than products associated with names which included back vowels [36]. Psycholinguistic research showed that a long distance between objects promoted speech production of *u* and *æ*, whereas a short distance between objects promoted speech production of *i* [37]. As another perspective of sound symbolism of vowels (vowel symbolism [32]), Hamano theoretically showed that vowel length was associated with temporal and spatial length of actions (i.e., speed and length [32]). Based on the vowel length, we can categorize sound-symbolic words, including long vowel(s) and sound-symbolic words, including short vowel(s). Vowel length in speech can be detected by infants [38,39], although the learning of vowel length consists of many factors that affect vowel length (e.g., vowel height [40]). In addition, another study suggested that 8- to 10-month-old infants preferred bi-syllabic heavy to light words which included stimuli with long vowels [41]. Although few psycholinguistic studies have examined the effects of vowel length in Japanese sound-symbolic words, a German psycholinguistic study conducted by Bross [42] reported that German pseudowords which included a long vowel (e.g., mutoh) were associated with long or large objects, while German pseudowords, which included short vowels (e.g., mutto), were associated with short or small objects. The author also showed that the associative relations between vowel length and object length were strong connections, whereas the associative relations between vowel length and object size were weak connections [42]. Although previous theoretical and experimental studies have shown that vowel length was associated with time (i.e., speed) and distance (i.e., length [32]) or size [42], vowel length would be also associated with other perceptual features (e.g., visual imageability, auditory imageability, and tactile imageability) and emotional features (e.g., familiarity, emotional valence, and arousal) based on previous psycholinguistic findings of vowel symbolism [14,36].

The aim of the current study is to investigate the effects of vowel length in written Japanese sound-symbolic words. In this survey, participants evaluated each written Japanese sound-symbolic word using eight semantic differential scales (familiarity, visual imageability, auditory imageability, tactile imageability, emotional valence, arousal, length, and speed) on a 5-point semantic differential scale from 1 to 5 (e.g., familiarity: 1: unfamiliar; 5: familiar). We selected six semantic differential scales from a behavioral study that examined written Japanese sound-symbolic words [28]. We also selected two semantic differential scales (length and speed) based on a theoretical study of Japanese sound symbolism [32] and an experiment that clarified the relationship between vowel length and object length [42]. The current study extended the previous studies by using selected semantic differential scales. A previous study successfully compared 10 sound-symbolic words which included two voiced consonants (e.g., *ダラダラ* in Japanese, *daradara*) with 10 sound-symbolic words which included two unvoiced consonants (e.g., *タラタラ* in Japanese, *taratara*) in order to identify sound-symbolic effects of voiced consonants [28]. Therefore, we compared 10 sound-symbolic words with two long vowels (e.g., *ブーブー* in Japanese, *buubuu*) with those with two short vowels (e.g., *ブブ* in Japanese, *bubu*) in order to identify sound-symbolic effects of vowel length using the successful methods of the previous study. The stimuli were written Japanese sound-symbolic words. Psycholinguistic studies have focused not only on the sound-symbolic phenomenon in European languages [3,5] but also on non-European languages, including the Japanese language [14,28]. The Japanese language also includes numerous sound-symbolic words [32]. For instance, some dictionaries only list Japanese sound-symbolic words [43]. In this study, the sample was native speakers of Japanese to assess Japanese native speakers’ evaluations of Japanese sound-symbolic words. Since previous studies on sound symbolism have focused on young samples’ evaluations of vowels and sound-symbolic words [14,28], we collected data from a wider range of samples from a crowdsourcing company and Google Forms rather than samples of previous research in order to control for age effects on sound-symbolic judgments. In addition, we pseudorandomized the presentation order of items between participants to control the presentation order effects of stimuli [44]. We predicted that written Japanese sound-symbolic words with long vowels would be perceived as spatially longer and temporally slower than those with short vowels. This prediction was consistent with previous theoretical and experimental findings [32,42]. A theoretical study showed that Japanese sound-symbolic words with short vowels showed events which were completed instantaneously (temporally speedy) and in a short distance (spatially short), whereas Japanese sound-symbolic words with long vowels showed events that took a long time (temporally slow) and space (spatially long) [32]. An experiment also reported that vowel length was spatially associated with object length [42]. In addition, we also predicted that the vowel length in written Japanese sound-symbolic words affects familiarity, multisensory imageabilities, emotional valence, and arousal. A previous study showed that each written Japanese vowel (*a*, *i*, *u*, *e*, and *o*) could be individually associated with specific multisensory features including physical aspects and emotional features including familiarity, emotional valence, and arousal [14]. Although the previous findings did not directly examine effects of vowel length to familiarity, multisensory imageabilities, emotional valence, and arousal, the previous results suggest that a written Japanese vowel itself could be differentially connected to familiarity, multisensory imageabilities, emotional valence, and arousal [14].

## 2. Materials and Methods

### 2.1. Participants

A total of 209 Japanese native speakers (115 females; *M_age_* = 39.42; *SD_age_* = 9.25; *age range* = 20–70 years) participated in this survey research. These participants were recruited from a crowd-sourcing company (Crowdworks, Inc., Tokyo, Japan). Each participant received 220 Japanese yen (JPY) as a monetary reward for their participation after completion of the survey. After each participant read and consented to the written explanation of the study, they answered the questionnaire. This research was approved by the ethical committee of the Graduate School of Humanities and Social Sciences at Hiroshima University (approval code: 2020001).

### 2.2. Materials

This study consisted of 20 Japanese sound-symbolic words (Appendix A). The sound-symbolic words were selected from a Japanese sound-symbolic dictionary [43], whereas two written Japanese sound-symbolic words (*hyohyo* and *nyanya*) checked by a native speaker of Japanese (the last author) were added. Based on a previous study that examined sound-symbolic effects of consonants by comparing 10 pairs of sound-symbolic words which included two voiced consonants (e.g., *ダラダラ* in Japanese, *daradara*) with sound-symbolic words which included two unvoiced consonants (e.g., *タラタラ* in Japanese, *taratara*) [28], we collected 10 pairs of sound-symbolic words which included two long vowels (e.g., *ブーブー* in Japanese, *buubuu*) and sound-symbolic words which included two short vowels (e.g., *ブブ* in Japanese, *bubu*). These written sound-symbolic words were shown in Japanese katakana moraic script, which is one of the traditional scripts of Japanese, alongside the morphographic kanji and moraic hiragana scripts [45,46]. The Roman script was also used in certain circumstances [45,46]. Katakana moraic script was used for loanwords (foreign words) and native words, which included sound-symbolic words, whereas kanji and hiragana scripts were used mainly for native words. A katakana symbol generally represented a pair of a consonant or a palatalized consonant and vowel or a vowel itself (e.g., a pair of a consonant and vowel: *ブ* in Japanese, *bu*; a vowel itself: *ア* in Japanese, *a*). Palatalization was represented in the orthography by attaching small *ya* (*ヤ*), *yu* (ユ), and *yo* (*ヨ*) to the preceding katakana or hiragana symbol (e.g., *チュ* in Japanese, *chu* [46]). Each long vowel of a pair of a consonant and vowel or a vowel itself was shown as *ー* (dash, *chōonpu* in Japanese; [46]) attached to a katakana or hiragana symbol in the Japanese writing system (e.g., *ブー* in Japanese, *buu*; [46]).

### 2.3. Procedures

We used Google Forms to conduct the survey. Before the survey, all participants read an explanation about the task. Participants were asked “Please evaluate presented onomatopoeic words by using eight evaluative items of five levels from one to five. The eight evaluative items of each onomatopoeic word consist of familiarity (1, unfamiliar; 5, familiar), visual imageability (1: not visually imageable, 5: visually imageable), auditory imageability (1: not auditorily imageable, 5: auditorily imageable), tactile imageability (1: not tactilely imageable, 5: tactilely imageable), emotional valence (1: dislike; 5: like), arousal (1: calm; 5: excited), length (1: short; 5: long), and speed (1: slow; 5: fast). Please choose the most appropriate number from 1 to 5 for each evaluative item.” The scales of the evaluative items were semantic differential scales [30]. These semantic differential scales were also based on theoretical linguistic and psycholinguistic research [13,14,28,32,47]. To control the order of the presentation of the stimuli, the order of the evaluative items was pseudorandomized between participants by preparing two lists of items to evaluate. We previously wrote a necessity to control the presentation order of written stimuli in surveys as a future direction of a previous article [14].

We used linear mixed-effects models to include participants and paired items as random effects, vowel length (0: SV; 1: LV) as fixed effects, and subjective evaluations of 5-point semantic differential scales (familiarity, visual imageability, auditory imageability, tactile imageability, emotional valence, arousal, length, and speed) as dependent variables [24,28,48]. Based on the linear mixed-effects models, we clarified the differences between the perceptual and emotional evaluations of LV and SV.

## 3. Results

Descriptive statistics were analyzed with the means and standard deviations. Cronbach’s alphas were calculated to assess reliabilities of semantic differential scales and a linear mixed-effects model analysis was conducted to examine the differences between subjective evaluations of LV and SV. First, we calculated means and standard deviations by using R [49] and a psych package [50] to assess the means and standard deviations of all words, LV, SV, and each written Japanese sound-symbolic word. We also checked whether the calculated means and standard deviations of R were consistent using Microsoft Excel and the statistical software SPSS. The means and standard deviations of subjective evaluations of all written Japanese sound-symbolic words, LV, and SV are shown in Table 1, whereas the means and standard deviations of each written Japanese sound-symbolic word are shown in Table S1.

Second, we calculated Cronbach’s alphas to examine the reliability of each subjective evaluation. Regarding written Japanese sound-symbolic words with long vowels, the Cronbach’s alphas (raw alphas in the psych package on R) for familiarity, visual imageabilities, auditory imageability, tactile imageability, emotional valence (affection), arousal (excitement), length, and speed were 0.73, 0.74, 0.78, 0.79, 0.72, 0.68, 0.89, and 0.75, respectively. Regarding written Japanese sound-symbolic words with short vowels, the Cronbach’s alphas (raw alphas in the psych package on R) for familiarity, visual imageability, auditory imageability, tactile imageability, emotional valence (affection), arousal (excitement), length, and speed were 0.77, 0.74, 0.79, 0.79, 0.72, 0.67, 0.83, and 0.87, respectively. Cronbach’s alphas were also assessed using SPSS statistical software. The Cronbach’s alphas for the semantic differential scales associated with evaluations excluding arousal were greater than 0.70. The semantic differential scales of arousal (0.68 in LV and 0.67 in SV, respectively) were at least higher than 0.65. In general, a Cronbach’s alpha of more than 0.70 is acceptable [51]. The Cronbach’s alphas of arousal in this study were categorized as arbitrary labels such as reasonable, adequate, moderate, and satisfactory as used in previous studies [52]. Published research shows that a Cronbach’s alpha of 0.6 or 0.7 is acceptable [53]. Thus, these findings suggested that the semantic differential scales would be approximately reliable, although the Cronbach’s alphas of arousal were not higher than 0.7 (0.68 in LV and 0.67 in SV).

Third, we conducted a linear mixed-effects model analysis to examine the differences between subjective evaluations of written Japanese sound-symbolic words, with long and short vowels. In the linear mixed-effects model, random effects were participants and paired items (paired words), and fixed effects (independent variables) were vowel length (0: SV; 1: LV). In addition, dependent variables were all the evaluations of the 5-point semantic differential scales such as familiarity, visual imageability, auditory imageability, tactile imageability, emotional valence, arousal, length, and speed. For the mixed-effects modeling, we used R [49], lme4 [54,55], and lmerTest packages [56]. Each syntax of the mixed-effects models was lmer (each subjective evaluation ~ vowel length + (1 | participant) + (1 | paired items), data = data, control = lmerControl (optimizer = “bobyqa,” optCtrl = list (maxfun = 100,000))) based on previous psycholinguistic research [24,28,48]. The results showed that familiarity, visual imageability, auditory imageability, tactile imageability, emotional valence, arousal, and length were higher for LV than SV, while speed was only higher for SV than LV (see Table 2).

## 4. Discussion

In this survey research, we investigated whether vowel length (long or short vowels) affected perceptual and emotional subjective evaluations of written Japanese sound-symbolic words. Two findings emerged from this survey: First, written Japanese sound-symbolic words with long vowels were perceived as more familiar, visually imageable, auditorily imageable, tactilely imageable, preferable, excited, and longer than those with short vowels. Second, written Japanese sound-symbolic words with short vowels were perceived as faster than those with long vowels. The current findings suggest that vowel length in written Japanese sound-symbolic words increases the perceptually and emotionally subjective evaluations of Japanese sound-symbolic words.

### 4.1. Effects of Vowel Length in Written Words

The current study showed that written Japanese sound-symbolic words with long vowels were perceived as more familiar, visually imageable, auditorily imageable, tactilely imageable, preferable, excited, and longer than those with short vowels. Our findings were congruent with previous findings. Hamano [32] theoretically showed that sound-symbolic words with a long vowel referred to spatially and temporally longer actions than those with a short vowel. In addition, Bross [42] showed that names with a long vowel were associated with longer and bigger objects than names with a short vowel. The current and previous findings suggest that the vowel length of sound-symbolic words in a language increases the temporal and spatial length of referents (actions and objects).

Alternatively, the results of the current research also showed that the vowel length of sound-symbolic words increased multisensory imageabilities, emotional valence, and arousal. Words which included long vowel(s) increased the perceptual and emotional referents of words which included sound-symbolic words. In fact, Pathak et al. reported that words which included long vowels were more associated with sweet food than words which included short vowels [57]. Another study showed that words which included long vowels negatively correlated with sharpness [58], although this finding does not suggest that words which included long vowels increased the perceptual and emotional referents of words. Taken together, words including long vowels could affect multisensory referents of words.

The perceptual and emotional sensitivity can also be orthographically affected by written sound-symbolic words. A previous study reported that initial consonants of written sound-symbolic words affected multisensory imageabilities, emotional valence, and arousal (e.g., *garigari* vs. *karikari* [28]). Cuskley and colleagues reported that written pseudowords with written round letters were associated with round figures, while pseudowords with spikey letters were associated with spiky figures [15]. Another study showed that written angular letters in spiky shapes as frames facilitated response times in a lexical decision task [16]. These previous findings suggest that visual properties of linguistic features (e.g., visual characteristics of letters and font styles) could be congruent with the visual properties of figures as referents (spiky or round figures [15]) or background (e.g., frames [16]). In this study, we used written Japanese sound-symbolic words which included long vowels (e.g., *フーフー*, *fuufuu*) and short vowels (e.g., *フフ*, *fufu*). The long vowels are represented as a dash (*ー*: *chōonpu* in Japanese [46]). The written form of the dash (*ー*: *chōonpu*) could affect the perceptually and emotionally subjective evaluations of written Japanese sound-symbolic words. Ando et al. (2021) reported that native speakers of Japanese evaluated that the written Japanese vowel *i* (*イ*) was thinner than other written Japanese vowels such as *a* (*ア*), *u* (*ウ*), *e* (*エ*), and *o* (*オ* [14]). In the previous case, the visual feature of the written Japanese vowel *i* (*イ*) might be perceived as thinner than the others. Similar to previous findings, in this study, participants might have perceived written Japanese sound-symbolic words, which included two long vowels shown with 2 dashes (e.g., *ブーブー* in Japanese, *buubuu*) as more familiar, visually imageable, auditorily imageable, tactilely imageable, preferable, excited, longer, and slower than written Japanese sound-symbolic words which included short vowels shown with no dash (e.g., *ブブ* in Japanese, *bubu*). As another perspective of the differences between written Japanese sound-symbolic words, including long and short vowels, character spacing might affect the evaluations. Previous research has shown that text spacing was essential for increasing reading performance, readability, and preference, as well as decreasing fatigue in reading [59,60]. In this study, since the two dashes (*ー*: *chōonpu*) in written Japanese sound-symbolic words made text spacing larger between characters, participants could easily perceive written Japanese sound-symbolic words which included two long vowels, which might increase the perceptual and emotional evaluations.

### 4.2. Future Directions

Future studies should develop these findings in terms of research methods. First, spoken sound-symbolic words were used as stimuli for the survey. If researchers use spoken sound-symbolic words as stimuli, they may need to control the auditory features of the stimuli (e.g., sound volume, sound pressure, and speakers). Second, researchers may directly use perceptual references including pictures (e.g., line drawings), sounds, objects [61], foods, or drinks [62] as the referents of sound-symbolic words in rating or matching tasks. The advantage of using perceptual referents is that participants can directly evaluate non-arbitrary relationships between written, spoken, or other forms of real words, including sound-symbolic words or pseudowords and perceptual or emotional referents. Alternatively, researchers may also use wh-questions or naming tasks as the tasks for the production of words or pseudowords [63,64,65,66,67]. Third, researchers can also examine how participants associate sound-symbolic words with referents by using associative learning methods. Researchers have investigated arbitrarily associative learning of meaningless or unfamiliar words in a native or second language with its referential features (meanings) [23,24,68,69,70], while other researchers have also examined non-arbitrarily associative learning of words and referents [17,18,19,20,21]. Finally, although we compared written Japanese sound-symbolic words including long vowels with those including short vowels, the subjective evaluations of the written Japanese sound-symbolic words might be affected by not only the vowel length, but also the meanings (referents) of the presented Japanese sound-symbolic words. In fact, the presented pairs of the written Japanese sound-symbolic words in this study include differential meanings (referents) associated with subjective evaluations measured in this study (see Appendix A), since we could not find pairs of written Japanese sound-symbolic words associated with the same meanings (referents). In sound symbolism, researchers have hypothesized that vowels and consonants in words directly affect referential features of sound-symbolic words in languages [3,5,28,32]. However, if researchers can find pairs of sound-symbolic words associated with the same or more similar meanings (referents), researchers may rigorously clarify effects of vowel length in sound-symbolic words by using the controlled stimuli and methods used in this study.

## 5. Conclusions

In this survey, in which Japanese native speakers subjectively evaluated each written Japanese sound-symbolic word, we examined the effects of vowel length in written Japanese sound-symbolic words by using eight semantic differential scales associated with familiarity, visual imageability, auditory imageability, tactile imageability, emotional valence, arousal, length, and speed. Two findings emerged from this survey. First, written Japanese sound-symbolic words with long vowels were perceived as more familiar, visually imageable, auditorily imageable, tactilely imageable, preferable, excited, and longer than those with short vowels. Second, written Japanese sound-symbolic words with short vowels were perceived as faster than those with long vowels. Taken together, these findings suggest that vowel length in written Japanese sound-symbolic words increases the perceptually and emotionally subjective evaluations of Japanese sound-symbolic words.

## Figures and Tables

**Table 1:** Descriptive statistics for written Japanese sound-symbolic words including long vowels (LV) and short vowels (SV).

Subjective Evaluations	ALL		LV		SV	
	*M*	*SD*	*M*	*SD*	*M*	*SD*
Familiarity	2.97	1.29	3.18	1.28	2.76	1.26
Visual imageability	2.85	1.40	3.20	1.40	2.49	1.31
Auditory imageability	3.14	1.39	3.52	1.33	2.77	1.35
Tactile imageability	2.44	1.32	2.69	1.36	2.19	1.22
Emotional valence	2.90	1.04	2.95	1.03	2.86	1.04
Arousal	2.65	1.06	2.71	1.10	2.59	1.00
Length	2.72	1.30	3.69	0.91	1.75	0.80
Speed	2.95	1.07	2.71	1.00	3.19	1.08

*M*: mean; *SD*: standard deviation; ALL: all written Japanese sound-symbolic words used in this study; LV: written Japanese sound-symbolic words including long vowels; SV: written Japanese sound-symbolic words including short vowels. These means and standard deviations were calculated in R [49] and psych [50]. In the survey study, participants evaluated each written Japanese sound-symbolic word by using 5-point semantic differential scales [29,30] associated with familiarity (1: unfamiliar; 5: familiar), visual imageability (1: not visually imageable; 5: visually imageable), auditory imageability (1: not auditorily imageable; 5: auditorily imageable), tactile imageability (1: not tactilely imageable; 5: tactilely imageable), emotional valence (1: dislike; 5: like), arousal (1: calm; 5: excited), length (1: short; 5: long), and speed (1: slow; 5: fast).

**Table 2:** Results of mixed-effects models for paired list of written Japanese sound-symbolic words including long vowels (LV) and short vowels (SV).

	F	VI	AI	TI	EV	A	L	S
**Random Effects**	**Variance (*SD*)**							
**Participants** **(intercept)**	0.24 (0.49)	0.25 (0.50)	0.32 (0.57)	0.34 (0.58)	0.17 (0.41)	0.12 (0.35)	0.08 (0.28)	0.13 (0.36)
**Paired items** **(intercept)**	0.42 (0.65)	0.45 (0.67)	0.39 (0.62)	0.32 (0.57)	0.25 (0.50)	0.27 (0.52)	0.02 (0.14)	0.04 (0.21)
**Residual**	1.00 (1.00)	1.17 (1.08)	1.13 (1.06)	1.04 (1.02)	0.69 (0.83)	0.75 (0.86)	0.64 (0.80)	0.91 (0.95)
**Fixed effects**	**Estimate (*SE*)**							
**Intercept**	2.76 (0.21) ***	2.49 (0.22) ***	2.77 (0.20) ***	2.19 (0.18) ***	2.86 (0.16) ***	2.59 (0.17) ***	1.75 (0.05) ***	3.19 (0.07) ***
**Vowel length**	0.43 (0.03) ***	0.71 (0.03) ***	0.75 (0.03) ***	0.51 (0.03) ***	0.10 (0.03) ***	0.13 (0.03) ***	1.95 (0.02) ***	−0.49 (0.03) ***

F: familiarity; VI: visual imageability; AI: auditory imageability; TI: tactile imageability; EV: emotional valence; A: arousal; L: length; S: speed; *SD*: standard deviation; *SE*: standard error; ***: *p* < 0.001. The mixed-effects models were conducted in R [49], R packages included lme4 [54,55] and lmerTest [56]. Dependent variables are subjective evaluations, whereas participants and paired words are random effects. In addition, vowel length (0: SV; 1: LV) is a fixed effect. In the survey study, participants evaluated each written Japanese sound-symbolic word by using 5-point semantic differential scales [29,30] associated with familiarity (1: unfamiliar; 5: familiar), visual imageability (1: not visually imageable; 5: visually imageable), auditory imageability (1: not auditorily imageable; 5: auditorily imageable), tactile imageability (1: not tactilely imageable; 5: tactilely imageable), emotional valence (1: dislike; 5: like), arousal (1: calm; 5: excited), length (1: short; 5: long), and speed (1: slow; 5: fast).

## Data Availability

The data are available on request to the corresponding author.

## Supplementary Materials

The following are available online at https://www.mdpi.com/article/10.3390/bs11060090/s1, Table S1: Descriptive statistics for written Japanese sound-symbolic words including long vowels (LV) and short vowels (SV).

## Appendix A

**Table A1:** Stimuli of Written Japanese Sound-Symbolic Words including Long Vowels (LV) and Short Vowels (SV).

LV		SV	
**Japanese Characters, Alphabetic Letters (Pronunciation)**	**English Meanings**	**Japanese Characters, Alphabetic Letters (Pronunciation)**	**English Meanings**
*フーフー, fuufuu*	breathing on something	*フフ, fufu*	giggling at something or somebody
*ジージー, jiijii*	buzz of a cicada; buzz of bell; sound of burning something	*ジジ, jiji*	buzz of an insect, squeak, or sound of burning something
*ヒーヒー, hiihii*	crying voice or behavior to hardship; bird song; throat sound in difficulty breathing; beeping	*ヒヒ, hihi*	sound of fast movement of arrow or wind; beeping
*シーシー, shiishii*	peeing; sound of peeing; getting out crumbs between teeth by using a toothpick	*シシ, shishi*	sobbing
*チューチュー, chuuchuu*	squeak; chirp; sucking something	*チュチュ, chuchu*	chirp; sucking something; muah
*ブーブー, buubuu*	sound of pig or car; state of dissatisfaction	*ブブ, bubu*	buzz of a bee
*ヒョーヒョー, hyoohyoo*	sound of a trumpet or flute	*ヒョヒョ, hyohyo*	sound or behavior of fast and light movement
*ニャーニャー, nyaanyaa*	meow	*ニャニャ, nyanya*	meow; acting like a cat
*ジャージャー, jaajaa*	flow of a large amount of something	*ジャジャ, jaja*	flow of a large amount of something
*コーコー, kookoo*	barking of a fox; crowing of a rooster; a faint sound heard from a distance	*ココ, koko*	screech of a monkey

LV: written Japanese sound-symbolic words including long vowels; SV: written Japanese sound-symbolic words including short vowels. The Japanese character was a katakana character that are one type of Japanese character [45,46]. English meanings do not reflect the degree of each evaluation (familiarity, visual imageability, auditory imageability, tactile imageability, emotional valence, arousal, length, and speed). We translated written Japanese sound-symbolic words in a dictionary of sound-symbolic words [43] to English meanings, whereas we originally added two Japanese sound-symbolic words including *hyohyo* and *nyanya* and translated them to English after checking meanings with a native speaker of Japanese (the last author).
