# Supplementary material for: Vowel Length Expands Perceptual and Emotional Evaluations in Written Japanese Sound-Symbolic Words
Source: Behav Sci (Basel). 2021 Jun 21;11(6):90. doi: 10.3390/bs11060090 (PMC8234476; doi:10.3390/bs11060090)
Supplement: Supplementary file 1 [file behavsci-11-00090-s001.zip › behavsci-1220053-supplementary.pdf]

behavioral sciences

MDPI

Table S1. Descriptive statistics for written Japanese sound symbolic words including long vowels (LV) and short vowels (SV).

Japanese characters, alphabetic letters, vowel length (Long Vowel: LV; Short Vowel: SV)	familiarity		visual imageability		auditory imageability		tactile imageability		emotional valence		arousal		length		speed	
	M	*SD*	*M*	*SD*	*M*	*SD*	*M*	*SD*	*M*	*SD*	*M*	*SD*	*M*	*SD*	*M*	*SD*
フーフー, *fuufuu*, LV	3.74	1.02	3.63	1.22	4.01	1.04	3.20	1.26	3.30	0.81	1.95	0.94	3.72	0.95	2.38	0.88
フフ, *fufu*, SV	3.38	1.20	2.63	1.31	2.91	1.40	2.11	1.17	3.32	1.00	1.88	0.82	1.65	0.66	2.99	1.03
ジージー, *jiijii*, LV	2.76	1.11	2.94	1.22	3.37	1.19	2.50	1.18	2.60	0.91	2.89	0.99	3.78	0.92	2.33	0.90
ジジ, *jiji*, SV	2.68	1.21	2.66	1.27	2.87	1.30	2.30	1.18	2.75	0.95	2.84	0.91	1.63	0.77	2.97	1.17
ヒーヒー, *hiihii*, LV	2.67	1.03	2.98	1.30	3.26	1.17	2.20	1.21	2.31	0.87	3.44	0.96	3.58	0.86	2.71	0.90
ヒヒ, *hihi*, SV	2.33	1.13	2.34	1.31	2.56	1.30	1.92	1.09	2.46	0.89	2.75	0.90	1.67	0.81	3.40	1.07
シーシー, *shiishii*, LV	3.03	1.14	2.79	1.31	3.23	1.27	2.34	1.18	2.79	0.84	2.31	0.86	3.65	0.88	2.70	0.89
シシ, *shishi*, SV	2.17	1.00	2.01	1.17	2.17	1.20	1.91	1.06	2.58	0.80	2.55	0.92	1.63	0.71	3.55	1.04
チューチュー, *chuuchuu*, LV	4.00	0.98	4.05	1.00	4.22	0.91	3.66	1.19	3.49	0.99	2.51	0.92	3.81	0.91	2.74	1.00
チュチュ, *chuchu*, SV	3.62	1.03	3.36	1.13	3.52	1.17	3.21	1.23	3.49	1.00	2.48	0.98	1.84	0.82	3.41	1.01
ブーブー, *buubuu*, LV	3.54	1.24	3.75	1.19	4.13	1.04	2.89	1.28	2.74	1.07	3.30	1.06	3.46	0.94	2.61	1.08
ブブ, *bubu*, SV	2.35	1.09	2.33	1.18	3.19	1.28	2.10	1.14	2.34	0.95	3.04	0.89	1.50	0.62	2.89	1.20
ヒョーヒョー, *hyoohyoo*, LV	2.25	1.07	2.13	1.12	2.46	1.24	1.88	1.06	2.52	0.88	2.78	0.94	3.68	0.85	3.01	1.03
ヒョヒョ, *hyohyo*, SV	2.26	1.16	1.89	1.10	2.19	1.16	1.66	0.93	2.47	0.92	2.53	0.85	2.00	0.93	3.20	1.05
ニャーニャー, *nyaanyaa*, LV	4.56	0.68	4.38	0.84	4.46	0.88	3.59	1.31	4.21	0.87	1.97	1.04	3.74	0.90	2.57	1.05
ニャニャ, *nyanya*, SV	3.81	1.05	3.60	1.16	3.52	1.23	2.88	1.34	3.59	1.12	2.14	0.93	2.08	0.90	3.15	0.98
ジャージャー, *jaajaa*, LV	3.05	1.18	3.57	1.28	3.89	1.16	3.05	1.31	2.81	0.85	3.58	0.98	3.89	0.89	3.36	1.00
ジャジャ, *jaja*, SV	2.18	1.02	2.13	1.09	2.53	1.22	1.97	1.04	2.39	0.81	3.53	0.91	1.93	0.83	3.20	1.01
コーコー, *kookoo*, LV	2.22	1.03	1.80	1.03	2.12	1.20	1.63	0.86	2.76	0.71	2.43	0.84	3.63	0.92	2.66	0.85
ココ, *koko*, SV	2.78	1.25	1.95	1.07	2.20	1.24	1.80	1.01	3.17	0.90	2.13	0.83	1.55	0.66	3.18	1.05

M: mean; *SD*: standard deviation; ALL: all written Japanese sound symbolic words used in this study; LV: written Japanese sound symbolic words including long vowels; SV: written Japanese sound symbolic words including short vowels. The Japanese character was a katakana character that are one of Japanese characters [45,46]. These means and standard deviations were calculated on R [49] and an R package psych [50]. In the survey study, participants evaluated each written Japanese sound symbolic word by using 5-point semantic differential scales [29,30] associated with familiarity (1: unfamiliar; 5: familiar), visual imageability (1: not visually imageable; 5: visually imageable), auditory imageability (1: not auditorily imageable; 5: auditorily imageable), tactile imageability (1: not tactilely imageability; 5: tactilely imageability), emotional valence (1: dislike; 5: like), arousal (1: calm; 5: excited), length (1: short; 5: long) and speed (1: slow; 5: fast). *fuufuu*, *fufu*, *jiijii*, *jiji*, *jaajaa*, and *jaja* could be written as *huuhuu*, *huhu*, *ziizii*, *zizi*, *zyaazyaa*, and *zyazya*, respectively.
